# Genome-wide characterization of genetic variants and putative regions under selection in meat and egg-type chicken lines

**DOI:** 10.1186/s12864-018-4444-0

**Published:** 2018-01-25

**Authors:** Clarissa Boschiero, Gabriel Costa Monteiro Moreira, Almas Ara Gheyas, Thaís Fernanda Godoy, Gustavo Gasparin, Pilar Drummond Sampaio Corrêa Mariani, Marcela Paduan, Aline Silva Mello Cesar, Mônica Corrêa Ledur, Luiz Lehmann Coutinho

**Affiliations:** 1 Animal Biotechnology Laboratory, Animal Science Department, Luiz de Queiroz College of Agriculture (ESALQ), University of São Paulo (USP), Piracicaba, SP 13418-900 Brazil; 2 Department of Genetics and Genomics, The Roslin Institute and Royal School of Veterinary Studies, University of Edinburgh, Easter Bush Campus, Midlothian, EH25 9RG UK; 3 Embrapa Suínos e Aves, Concórdia, SC 89715-899 Brazil; 4 Noble Reserch Institute, 2510 Sam Noble Parkway, Ardmore, Oklahoma 73401 USA

**Keywords:** Fat deposition, Fst, Genetic variants, Next generation sequencing, INDEL, Selection signatures, SNP, Poultry

## Abstract

**Background:**

Meat and egg-type chickens have been selected for several generations for different traits. Artificial and natural selection for different phenotypes can change frequency of genetic variants, leaving particular genomic footprints throghtout the genome. Thus, the aims of this study were to sequence 28 chickens from two Brazilian lines (meat and white egg-type) and use this information to characterize genome-wide genetic variations, identify putative regions under selection using Fst method, and find putative pathways under selection.

**Results:**

A total of 13.93 million SNPs and 1.36 million INDELs were identified, with more variants detected from the broiler (meat-type) line. Although most were located in non-coding regions, we identified 7255 intolerant non-synonymous SNPs, 512 stopgain/loss SNPs, 1381 frameshift and 1094 non-frameshift INDELs that may alter protein functions. Genes harboring intolerant non-synonymous SNPs affected metabolic pathways related mainly to reproduction and endocrine systems in the white-egg layer line, and lipid metabolism and metabolic diseases in the broiler line. Fst analysis in sliding windows, using SNPs and INDELs separately, identified over 300 putative regions of selection overlapping with more than 250 genes. For the first time in chicken, INDEL variants were considered for selection signature analysis, showing high level of correlation in results between SNP and INDEL data. The putative regions of selection signatures revealed interesting candidate genes and pathways related to important phenotypic traits in chicken, such as lipid metabolism, growth, reproduction, and cardiac development.

**Conclusions:**

In this study, Fst method was applied to identify high confidence putative regions under selection, providing novel insights into selection footprints that can help elucidate the functional mechanisms underlying different phenotypic traits relevant to meat and egg-type chicken lines. In addition, we generated a large catalog of line-specific and common genetic variants from a Brazilian broiler and a white egg layer line that can be used for genomic studies involving association analysis with phenotypes of economic interest to the poultry industry.

**Electronic supplementary material:**

The online version of this article (10.1186/s12864-018-4444-0) contains supplementary material, which is available to authorized users.

## Background

Chickens are one of the most important animals in the world, not only because of the intensive production of meat and eggs, but also because they are a model for developmental and genomic studies. The chicken genome has become an important tool for the worldwide avian research community since 2004, with the release of the first draft of the genome from a single female red jungle fowl, which is the wild ancestor of domestic chicken [1].

The domestic chicken has hundreds of different breeds, but commercial chickens can be divided into two main groups – broilers (meat-type) and layers (egg-type), which have been artificially selected for centuries. These two types have different phenotypic and genotypic profiles as a consequence of distinct genetic backgrounds and intensive genetic selection for different traits [2]. Broiler selection is focused on growth and muscle deposition, e.g. body weight, feed conversion, and breast weight. On the other hand, selection of layer chickens is focused mainly on reproductive traits, e.g. egg production and egg quality.

Different selection criteria applied for broiler and layer lines resulted in considerable differences in growth, development, and metabolic mechanisms during embryogenesis and hatching [3]. This intensive selection, which resulted in important changes in chicken phenotypes, can be detected by selection signatures in the genome [4, 5].

Recently, millions of SNPs and INDELs have been identified from different chicken genomes based on next generation sequencing data (NGS) [6–8], and it is now possible to detect regions of selection signatures on a genome-wide scale. Recent studies in chickens identified regions under selection using only broiler lines [9, 10], only layer lines [11, 12], or pooled sample data [4, 7].

In Brazil, Embrapa Swine and Poultry National Research Center maintains several chicken lines under multi-trait selection in Brazilian climatic and nutritional conditions for more than 20 years. Two of those lines, a white-egg type (layer) and a meat-type (broiler), due to considerable difference in their growth rates and carcass yields, were utilized to generate an F2 experimental population for QTL mapping studies [13, 14]. The CC layer line used in this study is a white-egg laying hen originated from the White Leghorn, and it has been selected since 1989 mainly for reproductive traits such as egg production and fertility, besides egg quality traits [15]. The broiler TT is a paternal line originated from the White Plymouth Rock, New Hampshire and White Cornish breeds, and it has been selected since 1992 mainly for growth and meat traits such as body weight, carcass, and cuts yields [15]. The average weight of TT broilers (~ 2.4 kg) was almost five-fold higher than that of CC layers (~ 0.5 kg), when reared as broilers, and also presented higher abdominal fat percentage and breast yield compared to CC [16]. Moreover, the layer line has the egg production average at 70 weeks of about 210 eggs and the first egg at 140 days [17]. Genomic characterization of these two lines and their comparison with existing commercial chicken lines can provide valuable information about artificial and natural selection in these lines.

In order to achieve a greater understanding of the genetic differences between these two Brazilian white-egg layer and broiler lines, a deep catalog of genetic variants (SNPs and INDELs) were generated by sequencing the genome of 28 chickens (14 per line) at medium sequencing coverage. SNPs and, for the first time in chicken, INDELs were utilized for detecting potential selection signatures using Fst approach. Genes located in candidate regions under selection and genes with variants of potential functional effect were further analyzed to consider their possible influence on economically important traits in chickens, such as fat deposition, growth and reproduction. In summary, this study provides novel insights into selection footprints that can help elucidate the functional mechanisms underlying important traits relevant to broiler and layer chicken lines. In addition, this study presents detailed information about variants in the Brazilian broiler and layer chicken lines that could be important for genetic studies involving association analysis with relevant phenotypes.

## Results

### Sequencing and alignment

Approximately 5 billion short reads were generated from 14 broilers, and 14 white-egg layers. After quality trimming, ~ 78% of the reads were retained. About ~ 98.8% of the quality-processed reads could be aligned to the chicken reference genome (Gallus_gallus4.0). After removal of PCR duplicates, the average sequencing coverage for the 28 individuals was ~ 11.2 X (average of 11.4 X for broiler line and 10.9 X for white-egg layer line).

### Polymorphism identification

The initial variant call resulted in 15,944,063 SNPs and 1,997,771 INDELs from 28 individuals analyzed, including autosomes 1–28 and 32, two linkage groups, mitochondrial, sex chromosomes (W/Z), and unplaced scaffolds. The genome-wide average density of variants considering variants from all 28 chickens was 16.84 SNPs/kb and 1.72 INDELs/kb. SNP densities for each line were 13.15 SNPs/kb for broilers and 10.82 SNPs/kb for layers. INDEL densities for each line were 1.41 INDELs/kb for broilers and 1.20 INDELs/kb for layers. Additional file 1 shows the frequency of substitution types in the SNPs that were initially called. The most frequent substitutions were G to A (18.5%) and C to T (18.4%).

Filtration of the variants based on several criteria (see Methods) removed around 4% of SNPs and 15% of INDELs, resulting in the final list of 13,927,521 SNPs and 1,361,946 INDELs from the 28 individuals. Unplaced scaffolds were not considered for the subsequent analyses, hence 478,182 SNPs and 26,057 INDELs were excluded from these regions. Also, for all the downstream analyses, only the filtered datasets of variants were considered.

The transition and transversion (TS/TV) ratio for SNPs initially detected in the whole-genome was 2.17, while the ratio in the filtered set was 2.31.

**Fig. 1:**
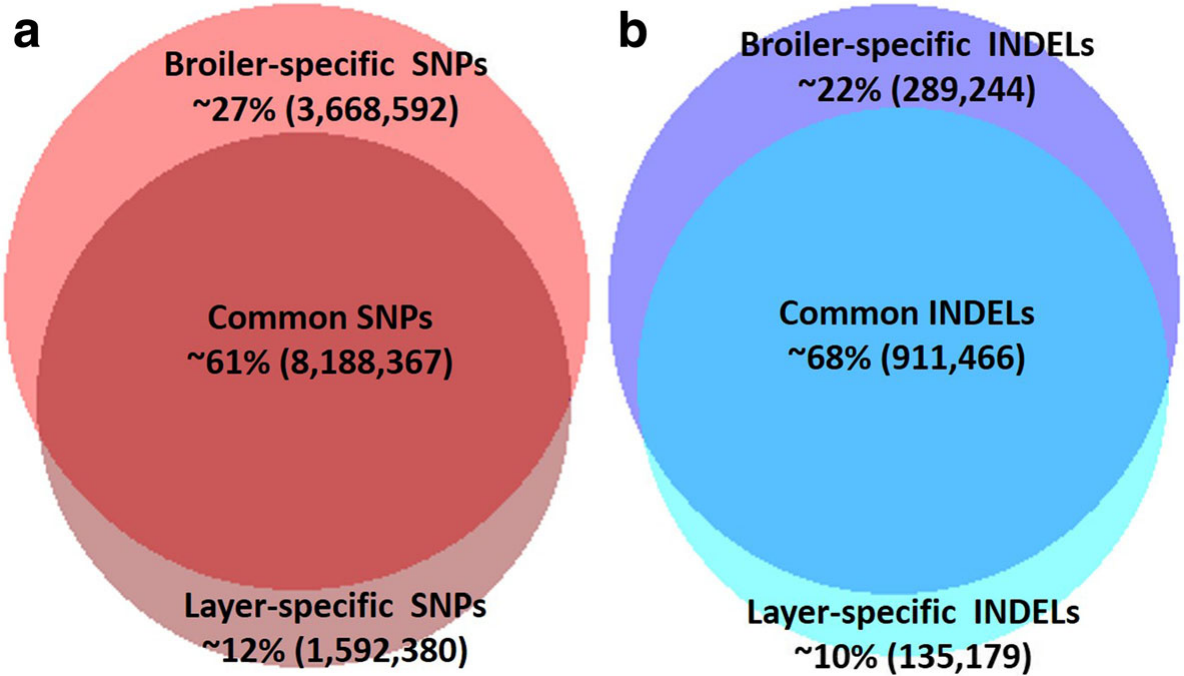
Venn diagrams of SNPs (**a**) and INDELs (**b**) shared between broiler and layer lines. The size of the circles reflects the relative number of filtered variants from each line within the total number of SNPs (*n* = 13,449,339) or INDELs (*n* = 1,335,889)

More variants were detected in the broiler (*n* = 11,856,959 SNPs and 1,200,710 INDELs in the filtered set) than in the layer line (*n* = 9,780,747 SNPs and 1,046,645 INDELs). About 26% (3,668,592) of all the SNPs were detected exclusively in the broiler line while ~ 12% (1,592,380) were detected only in the layer line (Fig. 1a). Similarly, about 22% (289,244) of the INDELs were specific to broiler, while ~ 10% (135,179) were specific to the layer line (Fig. 1b).

Moreover, a higher proportion of heterozygous variants were identified in the broiler line (on average 53.9% of SNPs and 46.6% of INDELs per individual) than in the layer line (average 44.8% of SNPs and 38.8% of INDELs) (Additional file 2). For both lines, INDEL variants showed greater level of homozygosity than SNPs. Considering all 28 chickens together from both lines, on average, 50.6% of the SNPs and 57.3% of the INDELs were homozygous per chicken, while the rest were heterozygous.

**Fig. 2:**
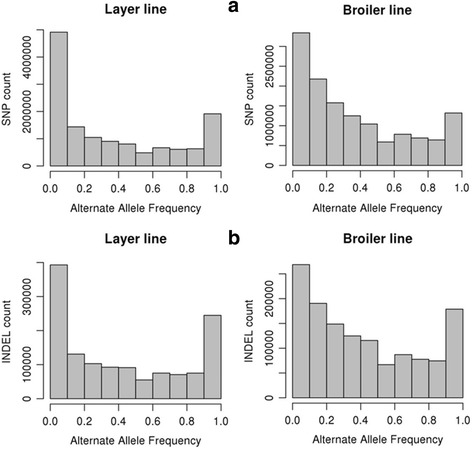
Distribution of alternative allele frequencies of SNPs (**a**) and INDELs (**b**) from layers and broilers

Frequency distributions of alternate allele (AAF) of SNPs and INDELs were estimated within the two lines. Our data shows that most of the SNPs (54%) and INDELs (46.3%) from the broiler and the layer lines had low frequency (≤ 0.3) (Fig. 2). However, some variants had reached near fixation (alternative allele frequency ≥ 0.9) within lines. About 9.6% of the SNPs and 8.8% of the INDELs in the layer line were fixed, while the broiler line showed 5.9% and 5.8% of the SNPs and INDELs, respectively, as fixed.

### Functional annotation of polymorphisms

Annotation showed that most of the variants belonged to non-coding regions of the genome, such as intergenic (38.5% SNPs and 39.4% INDELs), and intronic (42.1% SNPs and 42.9% INDELs) regions. Some of the variants (11.6% SNPs and 10.9% INDELs) had multiple annotations as those could be classified into multiple categories. Only about 1.2% of the SNPs were located in exonic regions, including synonymous (69.9%), non-synonymous (29.8%) and stop gain/loss SNPs (0.3%) (Table 1). Compared to SNPs, only 0.2% of the INDELs were located in exonic regions, including frameshift (54%) and non-frameshift INDELs (42.8%) (Table 1).

**Table 1:** Summary of functional annotation of SNP and INDEL variants from layer and broiler chicken lines

Category	SNP Count	SNP %	INDEL Count	INDEL %
Total no of variants	13,449,339		1,335,889	
Total annotation	15,223,947	100	1,500,714	100
Alternative annotation	1,774,608	11.66	164,825	10.98
Intergenic	5,866,525	38.53	590,736	39.36
Intronic	6,409,486	42.10	644,103	42.92
Exonic	175,258	1.15	2555	0.17
1 kb downstream	1,274,658	8.37	123,378	8.22
1 kb upstream	1,306,400	8.58	116,815	7.78
UTR3’	142,796	0.94	17,587	1.17
UTR5’	26,064	0.17	2462	0.16
Splicing	20,703	0.14	2866	0.19
ncRNA	1814	0.012	164	0.01
miRNA	243	0.002	48	0.003
Exonic categories				
Synonymous	122,502	69.90	–	–
Non-synonymous	52,219	29.80	–	–
Stopgain	424	0.24	–	–
Stoploss/retained	88	0.05	–	–
Frameshift	–	–	1381	54.01
Non-frameshift	–	–	1094	42.82
Coding unknown	25	0.01	80	3.13

In the present study, it was possible to predict the potential effect for 85% of the non-synonymous SNPs using the SIFT algorithm [18] which predicted 7255 SNPs (16%) to be evolutionary intolerant and 38,363 (84%) tolerant. Out of the 7255 intolerant SNPs, 2562 were present exclusively in the broiler line and 1302 were present only in the white-egg layer line.

### Metabolic pathway analyses of polymorphisms of potential functional impact

Metabolic pathways of genes harboring variants of high potential functional effects (e.g. intolerant SNPs and frameshift and non-frameshift INDELs) were investigated using the QIAGEN’s Ingenuity® Pathway Analysis software (IPA) [19].

The analysis of all genes containing intolerant SNPs from the white-egg layer and the broiler lines combined resulted in eight significant networks (Table 2). A total of 260 genes harboring intolerant SNPs participated in these eight pathways, which were related to connective and metabolic disorders, embryonic development, cardiovascular diseases, and carbohydrates metabolism, among others.

**Table 2:** Significant networks with intolerant SNPs from the layer and broiler lines combined

Molecules^a^	Total no of molecules	Score	Top diseases and functions
*AGAP3, APOLD1, CALCR, CDKL1, EDIL3, FHL5, LEPR, LEPROT, NCOR2, TGFBR1*	33	38	connective tissue disorders, metabolic disease, cellular assembly and organization
*AMOTL2, API5, BICD1, DZIP1, ERK, FBF1, HEATR1, IFT122, NIN, TEX11*	34	35	cellular assembly, organization, morphology and maintenance
*GALR3, GPR6, GPR20, GPR26, GPR97, GPR98, LPHN3, LYST, MC2R, MC3R*	34	35	cell signalling and interaction, cellular function and maintenance
*ABTB1, BRI3BP, CHD9, DDX18, DDX31, ESR1, FKBP7, GREB1, PPIL4, WHSC1*	32	33	organismal, embryonic and tissue development
*ACTR10, ANK2, APC2, DCTN4, DISC1, DNAH1, DNAH3, DNAH5, FARSB, KIF3C*	32	33	cellular assembly and organization, nervous system development, cancer
*ACTA2, DSC2, DSG2, DTX1, DTX3L, MTCL1, NOTCH2, SERPINB12, SRC, TMCO4*	33	33	cardiac arrhythmia, cardiovascular disease and congenital heart anomaly
*AK7, AK8, AK9, ARC, CAPN6, CAPN8, CAPN9, HIP1, KNTC1, PIPOX*	30	33	nucleic acid and carbohydrate metabolism
*AP5Z1, ASTE1, FLT3, FOXK2, HSP, HSPA2, HSPH1, METAP1, TSC1, PHF3*	32	31	cell cycle, developmental and hereditary disorders

^a^Example of ten molecules present in each network

When the same analysis was performed separately for the broiler and the white-egg layer lines using genes harboring intolerant SNPs specific to a line, clearly different networks were observed (Table 3). In the layer line, seven significant networks were identified mainly related to reproductive system development and cellular functions, and also endocrine system (Table 3). In the broiler line, 12 significant networks were identified, which were mainly related to lipid and carbohydrate metabolism, metabolic and dermatologic diseases, neural development, and also organism injury (Table 3).

**Table 3:** Top five significant networks from intolerant SNPs exclusively from the layer or broiler line

Molecules^a^	Total no of molecules	Score	Top diseases and functions
Layer line			
*BPI, DISC1, DYX1C1, IRG1, KNTC1, MAST2, MLKL, ODF2L, TELO2, TPO*	34	54	cellular morphology, assembly, organization and maintenance
*ACO1, FSH, ING1, LH, NEB, NPR2, PHKA2, RB1CC1, TNK2, Tropomyosin*	27	37	organ morphology, organismal development and reproductive system development
*AFF1, BARD1, CDC2, Cyclin A, Cyclin D, Cyclin E, EFHC1, ESR1, GNPAT, RB*	27	37	developmental disorder, immunological disease and cancer
*Alpha tubulin, Beta Tubulin, Dynein, EDIL3, ERK, FBN1, GPSM2, Growth Factor Receptor, INCENP, NPHP1*	26	35	cellular assembly, organization, function, maintenance and cycle
*ADCY, Caveolin, FHL5, GLUD1, IDH2, INSR, Insulin, LEPR, Proinsulin, Trypsin*	24	31	reproductive system development, cellular growth and proliferation and endocrine system disorders
Broiler line			
*AMY2A, Amylase, Apyrase, DMBT1, EDA2R, NGNL2, INF2, NKRF, NUAK2, OTULIN*	32	41	lipid metabolism, nervous system development and post-translational modification
*AMOTL2, ERK, FBF1, IQCE, MIPOL1, SCEL, SETD5, SH3RF2, SMPD1, TIPIN*	32	41	carbohydrate and drug metabolism and small molecule biochemistry
*AKAP, AKAP6, AKAP9, DISC1, LXN, MAPRE3, PCNXL4, PPM1E, RPS6KA1, SCT*	32	41	cellular morphology, assembly, organization, function and maintenance
*DCHS1, DHX30, EFCAB11, ESR, FTSJ3, HEATR1, MGA, MRPL3, NDC1, UTRN*	31	38	metabolic disease, hereditary disorder and cell cycle
*Aconitase, CRISP2, LAMA1, MEF2, MYBPH, MYH2, MYO9A, NFS1, OFD1, Tubulin*	30	37	cancer, dermatological diseases, organismal injury and abnormalities

^a^Example of ten molecules present in each network

The metabolic pathways obtained from the analysis of coding INDELs showed a total of 257 genes participating in nine metabolic pathways, mainly associated with diseases and disorders, for example: embryonic development, connective tissue disorders, gastrointestinal, metabolic, neurological and hepatic diseases, cardiovascular development, and cancer (Additional file 3). When the IPA analysis was performed separately for the broiler and the layer lines using genes harboring line-specific coding INDELs, three significant networks for each line were detected, and different networks were observed for the broiler and layer lines, e.g. growth and cardiac development in broilers (Additional file 4).

### Genome-wide scan for selection signatures

Fst values were obtained from SNP and INDEL datasets separately using an overlapping sliding window of 20 kb with 10 kb step size. A sufficient number of markers per window was obtained, ranging between 10 to 1094 SNPs (average of 268/window) and 5 to 114 INDELs (average of 26/window).

**Fig. 3:**
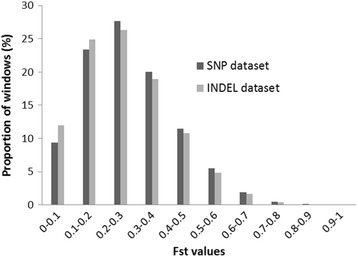
Genome-wide distribution of Fst in broiler and layer chickens from SNP and INDEL datasets. The weighted Fst distribution was obtained from 91,649 and 89,847 windows from SNP and INDEL datasets, respectively

Genome-wide weighted Fst distribution was examined (Fig. 3) based on 91,649 and 89,847 windows from SNP and INDEL separate datasets, respectively, between two lines (meat and white egg-type). The majority of the windows (60–63%) had low Fst values (< 0.3), and a few windows showed extremely high Fst values > 0.9 (11 and 3 windows for the SNP and INDEL datasets, respectively). SNP dataset had the mean weighted Fst of 0.2779 (SD 0.14), and INDEL dataset had the mean weighted Fst of 0.2645 (SD 0.14).

Even though the number of INDELs per window was smaller than those in the SNP dataset, similar regions were identified in the SNP and INDEL analyses, with a significant positive correlation (*r* = 0.66; *p* < 0.001) between the Fst values from the same window of putative selection signatures (top 1% Fst values) of SNPs and INDELs analysis.

**Fig. 4:**
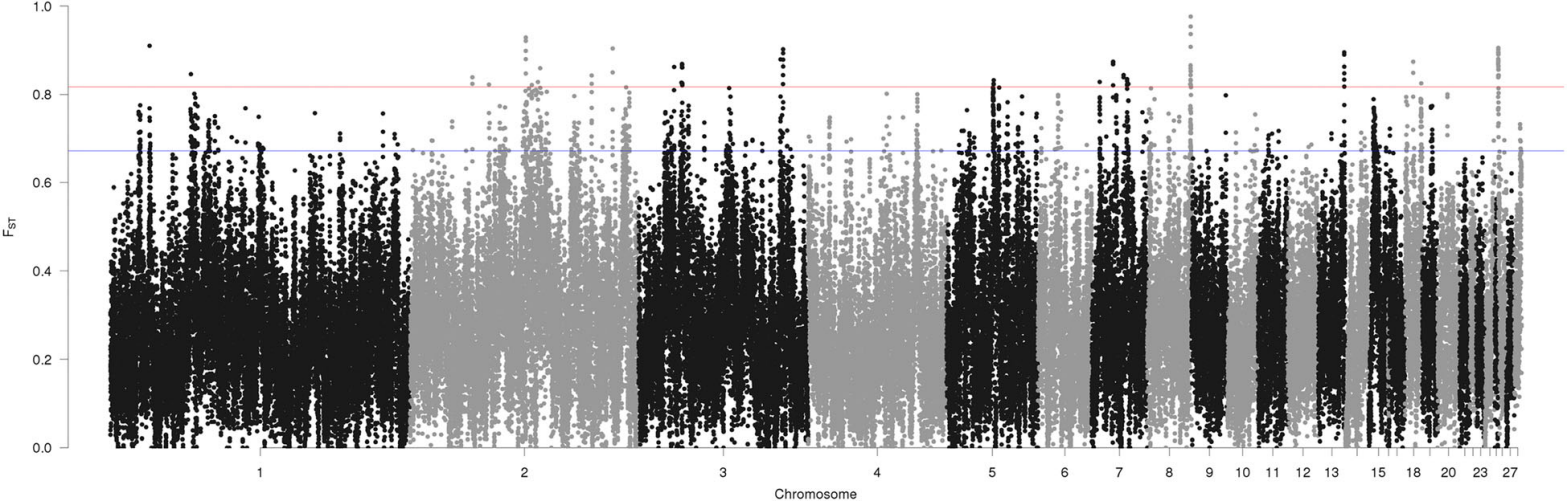
Manhattan plot of genome-wide putative signature selection regions between broiler and layer lines (SNP dataset). Blue line represents the top 1% of Fst values and red line represents the 0.1% of the Fst values

Based on the SNP dataset, 92 windows with top 0.1% Fst value (Fst ≥ 0.817, Fig. 4) were considered as strong candidates of selection signatures while 916 windows with top 1% Fst (Fst ≥ 0.6718, Fig. 4) were deemed as putative. These putative selection signature windows represented most of the chromosomes evaluated, except GGA16, 21, 22, 23, 25 and 27. The highest Fst value observed was 0.976 (GGA8: 28,220,001–28,240,000). Many windows that passed the top 1% Fst threshold could be merged into larger regions when those were adjacent or overlapping, e.g. on GGA26, 32 windows passing the threshold could be grouped into two regions only (GGA26:10,001–350,000 and GGA26:4,090,001–4,110,000). Merging adjacent windows resulted in 345 regions (~ 12.8 Mb in total) for SNP based analysis. The 916 putative selection signature regions from SNP-based analysis intersected with 307 genes, including 37 novel chicken genes, eight miRNAs, and four ultra-conserved elements (Additional file 5).

**Fig. 5:**
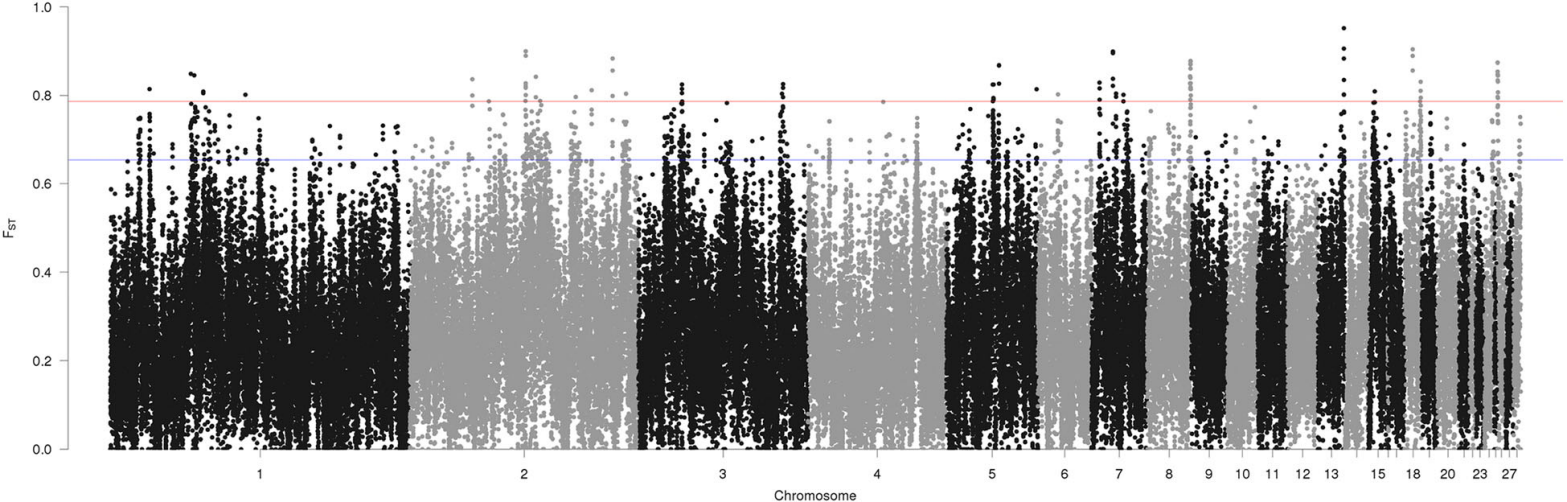
Manhattan plot of genome-wide putative signature selection regions between broiler and layer lines (INDEL dataset). Blue line represents the top 1% of Fst values and red line represents the 0.1% of the Fst values

When the INDEL dataset was analyzed, 90 candidate windows were identified with strong evidence of selection (top 0.1%, Fst ≥ 0.7865, Fig. 5), and 896 windows as putative selection signatures (top 1%, Fst ≥ 0.6537, Fig. 5). These regions represented all autosomes analyzed (chromosomes 1–28), except GGA12, 16, 22, 23, 25 and 27. The highest Fst value (0.951) was observed in one window on GGA13 (16,930,001–16,950,000). Merging the adjacent windows from INDEL-based analysis resulted in 425 regions (~ 13.5 Mb in total length). Annotation of these INDEL-based selection signatures represented 363 genes or functional features, including 48 novel chicken genes, nine miRNAs, two small nucleolar RNAs, and four ultra-conserved elements (Additional file 5).

Out of the 307 genes from the SNP-based analysis and the 363 genes from the INDEL-based analysis, 220 genes were common. When all merged signature regions (top 1% of Fst values) obtained from both analyses (345 and 425 regions) were compared, most of the regions (*n* = 260 with total length ~ 8.6 Mb) were found to be common between the two analyses.

### Candidate genes under selection for fat deposition and muscle development

A number of genes potentially related to fat deposition and muscle development were identified among the genes overlapping selection signature regions (Table 4).

**Table 4:**
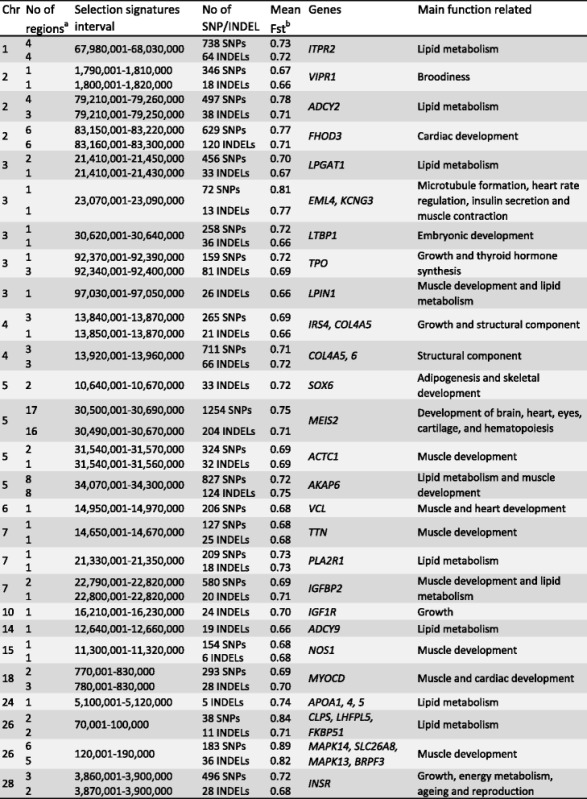
Genes associated with traits of interest in poultry located in putative signatures of selection regions

^a^Number of signature selection regions (windows) identified

^b^Mean value of Fst considering all windows combined

We analyzed all the genes under putative selection, 307 from SNP-based analysis and 363 from INDEL-based analysis, with GeneMANIA prediction server [20], which performs a functional enrichment analysis to find the biological functions of genes. The genes from the SNP-based selection signatures resulted in two enriched pathways: muscle system process and negative regulation of ion transport (Additional file 6). The 13 genes from the pathway related to muscle development were *ACTC1, AKAP6, ATP2A2*, *CACNA1S, CAV3, KCNMA1, MYOCD, NOS1, PDE4D*, *TPM4, TTN, VCL,* and *VIPR1*.

The genes from the INDEL-based selection signatures resulted in six enriched pathways related to lipid metabolism, e.g. regulation of lipase activity, and lipoprotein particle remodelling (Additional file 7). Some of the genes present in the pathways related to lipid metabolism were *AKAP6, APOA1/4/5, ADCY2/9, ITPR2, PLA2R1, SCARB1,* and *OPHN1*. Also, we identified one pathway related to muscle differentiation with genes that were also present in the SNP enrichment analysis, such as *ACTC1, AKAP6, NOS1, MYOCD,* and *TTN*.

Thus, candidate genes for fat deposition from SNP-based selection signature analysis include *ITPR2* (GGA1), *ADCY2* (GGA2), *LPGAT1* (GGA3), *AKAP6* (GGA5), *IGFBP2* and *PLA2R1* (GGA7), and *CLPS* (GGA26)*.* Candidate genes identified for muscle development are *ACTC1* and *AKAP6* (GGA5), *IGFBP2* (GGA7), *NOS1* (GGA15), *MYOCD* (GGA18), and *MAPK13/14* (GGA26).

All the above mentioned candidate genes for fat metabolism were detected by both SNP and INDEL-based analyses, with the addition of five more candidates detected only by INDEL analysis: *LPIN1* (GGA3), *ADCY9* (GGA14), and *APOA1/4/5* (GGA 24). For muscle development, the same candidate genes were identified in SNP and INDEL analyses, except the *MYOCD* gene, detected only in the SNP analysis, and *IGF1R*, detected only in the INDEL analysis.

Furthermore, we also checked genes containing any intolerant SNP that overlapped with selection signature regions. Sixty-three intolerant SNPs were found within selection signature regions, and they represented 39 genes (Additional file 8). Some of these genes have relevant functions, such as related to growth, e.g. *AKAP6*, *NOS1*, *MAPK13,* and *CACNA1S.*

We also identified candidate genes within the putative signature selection regions for other important traits in chicken such as cardiac, skeletal and embryonic development, broodiness, energy metabolism, and reproduction (Table 4). These candidates are described in more details in the Discussion section.

### Overlap of selection signatures with known QTLs

In order to to achieve greater accuracy in the detection of the putative selection signatures, the merged regions (345 and 425 from SNP and INDEL analyses, respectively) were compared with known QTL regions in chicken according to the Animal QTLdb (release 29, *n* = 5462) [21]. About 97% of the selection signature regions overlapped with one or more QTLs for different traits.

**Fig. 6:**
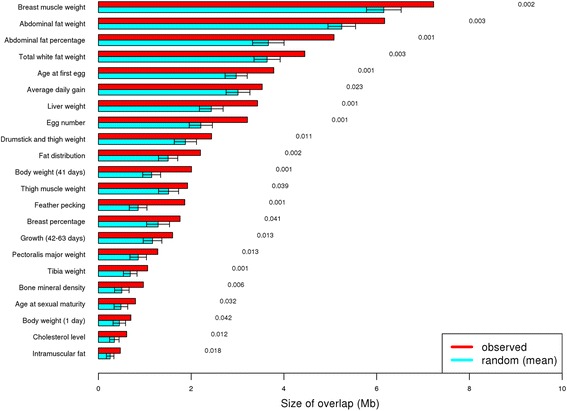
Significant overlap of putative selection signature regions from SNP-based analysis with relevant chicken QTL-traits. Red bars indicate the observed overlap (selection signature regions overlapping with the respective trait), blue bars indicate the random overlap (1000 permutations), and the error bars indicate the standard deviation. The permutation *p*-values are listed on the right

**Fig. 7:**
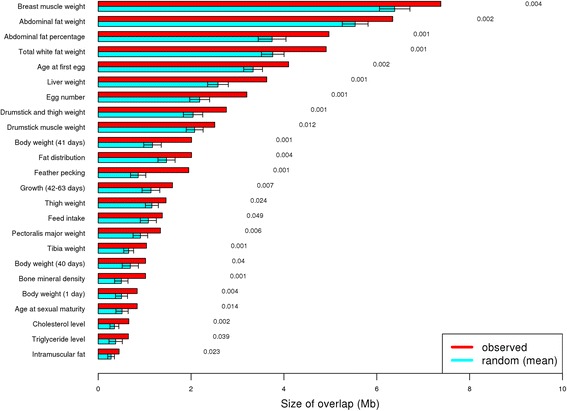
Significant overlap of putative selection signature regions from INDEL-based analysis with relevant chicken QTL-traits. Red bars indicate the observed overlap (selection signature regions overlapping with the respective trait), blue bars indicate the random overlap (1000 permutations), and the error bars indicate the standard deviation. The permutation *p*-values are listed on the right

Also, a statistical test based on permutation sampling was used to access the significance of the observed overlaps between the putative selection signature regions and QTLs. Based on the regions under selection from the SNP analysis, traits related to fat deposition, body weight, muscle weight, growth, egg production, bones, and others showed significant overlaps (permutation *p*-value < 0.05, Fig. 6). When the INDEL-based analysis was considered, similar traits had significant overlaps (Fig. 7).

### SNP and INDEL validation

SNPs were validated by comparison against two different datasets: (i) 15 M SNPs detected in a previous study by Gheyas et al. [7], and (ii) 600 K Affymetrix® Axiom® HD genotyping array data from two chickens (one male white-egg layer and one male broiler), which have also been sequenced in the present study. Comparison of the SNP catalog from Gheyas et al. [7] showed that 79.7% of our variants were common between the studies when only the genomic positions were compared, and 78.94% were common when both the position and allelic information were compared.

Comparison of the sequence genotype data against the 600 K SNP array dataset showed very high level of concordance (Additional file 9). For the two chickens used in this comparison, we could analyze genotypes of 575,599 SNPs for the layer (443,350 homozygous and 132,249 heterozygous) and 574,482 SNPs for the broiler (404,763 homozygous and 169,719 heterozygous), as these were present in the chip. Over 98% of the SNPs had the same genotypes between the NGS and array datasets (GEN-CONC), both for the broiler and the layer chicken. Slightly higher GEN-CONC was observed for homozygous SNPs than for heterozygous ones.

In addition, we randomly checked some of the discordant genotypes, and noticed that the majority of these had poor genotype quality (< 30) in NGS even though their variant qualities were high. The error rate in SNP genotype call (considering the non-concordance of genotypes between NGS and array data for SNPs that could be compared) was only 1.51% for the layer chicken and 1.81% for the broiler.

We also validated a large number of functional variants - intolerant non-synonymous and stopgain/loss SNPs against the 600 K array data. These included 757 intolerant SNPs (380 from layer and 377 from broiler chickens), and 37 stopgain/loss SNPs (18 from layer and 19 from broiler chickens). Importantly, over 41% of the 63 intolerant or stopgain/loss variants detected in the selection signature regions were present in the 600 K genotyping array and could be validated.

The INDELs identified in this study (*n* = 1,335,889) were also validated by comparison with results from two major recent studies [8, 22]. We combined these two previous datasets, and based on positions only, we observed that 77.24% (*n* = 1,031,932) of the INDELs in our study were common with the previous studies. When the alleles were also compared along with positions, 62.62% of the INDELs were validated (*n* = 836,552).

## Discussion

### Polymorphism identification and functional annotation

A genome-wide SNP and INDEL identification in the Brazilian white-egg layer (CC) and broiler (TT) lines was performed to obtain a detailed map of genetic variation in these lines. The initial variant call resulted in approximately 16 million SNPs and 2 million INDELs with an average density of 16.84 SNPs/kb and 1.72 INDELs/kb. Our results are consistent with recent studies, which reported similar densities in chicken [6–8, 23].

After filtration, a total of 13.93 million SNPs and 1.36 million INDELs were retained, with more variants detected from the broiler line, including heterozygous variants.

The transition and transversion (TS/TV) ratio for SNPs increased after the filtration process, showing that SNP filtration improved the TS/TV ratio, as higher ratios indicate better accuracy and lower false-positive rates [24]. In chicken, similar TS/TV ratios were reported [25].

Heterozygous variants are more difficult to be identified than homozygous ones. It has been estimated that a sequencing coverage of 6–10 X is sufficient to detect 99% of all variants (homozygous and heterozygous); however, some studies reported that a higher coverage (> 20 X) is necessary for detecting 99% of heterozygous SNPs [26, 27].

A recent study [28] analyzing the same Brazilian layer and broiler lines using reduced-representation-sequencing also reported a higher proportion of heterozygous SNPs in the broiler line. The greater variability and heterozygosity observed in the broiler line was likely due to its broad genetic background, which is similar to other commercial chickens and includes the White Plymouth Rock, New Hampshire and White Cornish breeds [15]. On the other hand, the layer line utilized in this study is a white-egg laying hen, which originated solely from the White Leghorn breed [15].

Even though there are millions of SNPs and INDELs along the genome, only a few are expected to explain the phenotypic differences observed between the two lines. As expected, annotation of the variants showed that most of them belonged to non-coding regions of the genome, and just 1.2% of the SNPs and 0.2% of the INDELs were located in exonic regions, including 122,502 non-synonymous SNPs. Non-synonymous SNPs and SNPs located in regulatory regions are known to have the highest phenotype impact [29].

Although, most of the variants were located in non-coding regions, we identified 7255 intolerant non-synonymous SNPs, 512 stopgain/loss SNPs, 1381 frameshift and 1094 non-frameshift INDELs that may alter protein functions.

Both frameshift and non-frameshift INDELs affect protein sequence by being located within coding regions. Frameshift mutations, however, can also have a major deleterious impact on protein function as these alter the amino-acid reading frame. On the other hand, non-frameshift INDELs do not change the reading frame, but they can still alter the protein function by inserting or deleting one or more amino-acids in the protein sequence. The impact of INDELs on transcription has not been well investigated, but it has been suggested that this may be considerable [30]. Non-coding INDELs, e.g. located in 1 kb down/upstream regions*,* can affect gene regulation by modulating transcription or silencing genes [30].

The chicken genome is still poorly annotated for functional non-coding regions [31], which can be involved in important biological functions as was shown in humans [32]. Because of this, it is currently difficult to predict the potential effect of variants located in non-coding regions, particularly in potentially important regions e.g. UTR, up/downstream, splicing and ncRNA.

### Metabolic pathway analyses of polymorphisms of potential functional impact

In order to gain insights into how the polymorphisms identified could explain the phenotypic changes observed in the two Brazilian chicken lines, metabolic pathways of genes harboring variants of high potential functional effects were investigated.

The analysis of all genes containing intolerant SNPs from the white-egg layer and the broiler lines combined resulted in eight networks related mainly to connective and metabolic disorders, embryonic development, cardiovascular diseases, and carbohydrate metabolism.

The effects of the potential disruptions of these important pathways caused by intolerant SNPs may explain various disorders observed in commercial broiler and layer chickens. Broiler chickens, for instance, have been selected for weight gain, feed efficiency, breast and carcass yields, which results in a substantial increase of growth in a short period. This particular scenario for meat-type chickens can result in heart ventricular hypertrophy and overload of the cardiopulmonary system, leading to pulmonary hypertension syndrome (ascites), which can cause chicken mortality or whole carcass condemnation during slaughter [33].

On the other hand, layer chickens, which have been mainly selected for high egg production, have a reduced body weight than the broiler chickens and can develop diseases such as fatty liver hemorrhagic syndrome (FLS) and osteoporosis [3]*.* FLS is a result of excessive accumulation of fat in the liver when lipoprotein transport is disrupted during high egg production and cause haemorrhage and sudden death [34].

When the analysis was performed using genes harboring intolerant SNPs specific to a line, clearly different networks were obtained for each line. In the layer line, networks were identified mainly related to reproductive system development, cellular functions, and the endocrine system. The latter in layers is particularly important for reproductive aspects, which involve different hormones and their receptors e.g. growth hormone (GH), prolactin (PRL), thyroids, luteinizing hormone (LH), melatonin, and others [35, 36]. For instance, GH is a regulator of ovarian and oviductal functions in chicken, acting in the modulation of LH and the synthesis of insulin-like growth factors [35]. Also, prolactin is involved with different physiological processes that are important for poultry reproduction; hence, *PRL* is a candidate gene for egg production in chicken [36].

In the broiler line, networks were obtained mainly related to lipid and carbohydrate metabolism, metabolic and dermatologic diseases, neural development, and organism injury. It is well known that commercial broiler lines gain much more weight and muscle mass than layer chickens. It was observed that at 41 days of age, the Brazilian broiler chicken line weighed on average 2395 g, while the layer chicken line weighed only 513 g, when reared as broilers, resulting in about a five-fold difference between these two lines. Also, the breast yield for the broiler line was 6% higher compared to the layer line [15, 16]. This difference in muscle development and growth between broilers and layers can be observed in the early stages of chicken embryos as previously reported [3]*.*

Another important difference between these two lines is lipid metabolism and fat deposition. Broiler chickens accumulate more fat than layers, and lipid metabolism differences were detected during chicken embryogenesis, e.g. higher triglyceride content in the liver of broilers [3]. It was observed that at 41 days of age, the Brazilian broiler chicken line presented 2.41% of abdominal fat percentage, while the layer chicken line displayed 0.16% [16]. However, so far, the differences between broiler and layer chicken lines regarding adipose growth are not well understood [37].

Frameshift and non-frameshift INDELs are very important as well, because they are located in coding regions, and both can affect protein sequence. The metabolic pathways obtained from the analysis of coding INDELs showed pathways mainly associated with diseases and disorders. Many diseases in humans have been found to be associated with INDELs (e.g. different types of cancer), and it was estimated that INDELs are responsible for 24% of the inherited diseases in humans [38]. Previous studies in chicken have also found INDELs to be associated with various health-related problems, such as retinal degeneration and embryonic mortality [39], production traits, such as egg production [40], and performance and carcass traits [41]. The frameshift and non-frameshift INDELs identified in the present study could also be involved with health problems, but further association studies need to be performed to elucidate their effects.

### Genome-wide scan for selection signatures

Intensive artificial selection can cause high degree of genetic differentiation between populations in specific genomic regions, which can result in selection footprints. There are different methods and tools available to detect evidence of selection. Traditionally, the Fst method has been a suitable choice based on allele frequency between populations [5].

Fst values were obtained from SNP and INDEL datasets separately using an overlapping sliding window of 20 kb, which was also utilized in previous studies in chicken [42, 43]. Although some studies used 40 kb windows [4, 7, 44], we decided to use 20 kb size-windows because previous studies reported that haplotype blocks in chicken have around 10 kb. The haplotype blocks described in chicken have different sizes depending on the line analyzed. In one study, haplotype block size was different in commercial and non-commercial broiler and layer chickens, but the length was typically less than 10 kb [42]. Also, traditional and village chickens were evaluated, and a median block size of 11–12 kb was observed [43]. Another reason to use 20 kb windows was to obtain a better resolution of the regions combined with a sufficient number of variants in each window.

We obtained a sufficient number of markers per window with an average of 268 SNPs/window and 26 INDELs/window. In similar studies in chicken, Qanbari et al. [44] obtained a mean of 199 SNPs per 30–40 kb window; Stainton et al. [10] had < 30 SNPs per window, and Gholami et al. [11] analyzed windows of 40 SNPs. Detecting regions under selection with Fst methods requires at least 10 samples [45]. Moreover, presence of a large number of markers significantly increases the power of the analysis [45]. Even though we only analyzed 14 individuals per line, our method is robust as NGS data were used with dense marker sets.

A majority of the windows had low Fst values (< 0.3), although a few windows showed extremely high Fst values (> 0.9). Our results corroborate with similar Fst-based studies in chicken. For instance, Gholami et al. [11] obtained Fst values using the Wright method in commercial layer chickens and observed average values, depending on the breed, between 0.09 and 0.27. Stainton et al. [10], like in our study, utilized the Weir and Cockerham’s pairwise Fst estimator and obtained mean values between 0.015 and 0.17 depending on the broiler line analyzed, reflecting the fact that each broiler line had slightly different selection criteria which resulted in a range of broiler lines with different characteristics.

Separate Fst analyses, with SNPs and INDELs, were performed to check if different putative signature regions would be detected. Most of the signature regions identified were found to be common between the two analyses (SNPs and INDELs), with a high level of correlation. This shows that even though the INDEL dataset was only ~ 10% of the SNP dataset in size, both datasets detected practically the same regions under selection. Our study is the first in chicken to use INDEL variants to detect footprints of selection. There are, however, a few studies in other species, for example in humans [46]. In this study, INDELs have been used for identification of selection signatures, and they suggested that regions surrounding INDELs are more frequently involved in recent selective sweeps [46]. We believe that INDEL variants are also a good option for selection signatures analyses even with a reduced density than SNPs, but further studies are necessary to evaluate it in more detail.

Genetic drift is a well-known factor that can also cause divergences in genomic regions. It is almost impossible to determine if a putative selection signature was caused by drift [47]. Besides, a combination of genetic drift and selection could be responsible for eliciting signals similar to selection footprints [48]. Despite that, there are different ways to minimize the false discovery of selective sweeps, such as the use of a stringent Fst cut-off or checking the overlap of those regions with QTLs [47, 49]. In our study, different steps were utilized to minimize the detection of false positive regions due to drift: (i) stringent Fst cut-off was applied (> 99% Fst); (ii) windows with less than 10 SNPs or 5 INDELs were excluded and weighted Fst values were used; (iii) putative selection signature regions were overlapped with known QTL regions from chicken QTLdb and permutation tests were applied to check the significance of these overlaps (results presented and discussed in sections below); (iv) putative selection signature regions were further interpreted based on different downstream analyses such as annotation and pathway enrichment analysis of genes within the signature regions (presented and discussed in sections below); and (v) Fst analyses were performed utilizing SNP and INDEL datasets separately to obtain more accurate results.

### Candidate genes under selection for fat deposition and muscle development

We analyzed all genes under putative selection using network and gene enrichment analyses, and gene function by literature search, and we identified candidate genes for fat deposition and muscle development. There were 307 from SNP-based analysis and 363 from INDEL-based analysis. Candidate genes associated with fat deposition were *ADCY2/9, APOA1/4/5, AKAP6*, *CLPS, IGFBP2, ITPR2, LPGAT1, LPIN1,* and *PLA2R1.* Candidate genes associated with muscle development were *ACTC1, AKAP6*, *IGFBP2*, *IGF1R*, *NOS1*, *MAPK13/14* and *MYOCD*.

The *AKAP6* gene is present in both muscle development and lipid metabolism pathways. A recent study suggests that this gene is an important regulator of muscle regeneration and myoblast differentiation in mice [50], in addition to its role in cardiac functions [51]. This gene seems to play a major role in the regulation of different processes, and further studies in chicken are necessary to investigate its role in greater details.

The 30 kb selective sweep region (GGA7:22,790,001–22,820,000), covering the gene *IGFBP2* identified in our study (with variants mainly fixed in the layer line), was previously reported in a comparison of various layer chickens [11]. This gene is known to be related to growth [52] and fat deposition [53] from previous studies in chicken.

A putative selection signature region of 20 kb size was identified (GGA10: 16,210,001–16,230,000) with 24 INDELs overlapping the gene *IGF1R* with variants mainly fixed in the layer line. Recent studies in chicken, using the same method described here, also identified the same gene under selection in a commercial broiler [10] and layer [12] lines. This gene plays an important role in chicken growth [54] and may also be related to reproduction in layers [55].

One selective sweep of 40 kb (GGA28: 3,860,001–3,900,000) overlapping the *insulin receptor* gene (*INSR*) was identified in our study with variants mainly fixed in the broiler line (Table 4). This region has been previously reported in studies with layers [44], broilers [4], and both broilers and layers [7]. Also, *INSR* was associated with growth in chicken in a previous study [56].

### Candidate genes under selection for other important traits in poultry

Apart from fat deposition and muscle development traits, genes within the putative signature selection regions were found to be candidates for other important traits in chicken, such as cardiac, skeletal and embryonic development, broodiness, energy metabolism, and reproduction. For instance, the *MEIS2* gene is related to the development of brain, heart, eyes, cartilage and hematopoiesis [57]. In our study, 17 adjacent windows were detected as putative selection signatures (GGA5: 30,490,001–30,690,000) harboring the *MEIS2* gene, which were merged into one large region of 200 kb, possibly indicating a strong selection region. In the study of Gheyas et al. [7], they identified broiler-specific SNPs in the *MEIS2* gene.

In addition, two genes, *ITPR2* and *VIPR1,* were identified in the putative selection signature regions, which are possibly related to layer reproductive traits*.* One recent GWAS study in chicken revealed that the *ITPR2* gene was associated with eggshell ultrastructure [58]. On the other hand, the *VIPR1* gene was found to be associated with broodiness [59], and egg number in chicken [60].

Certain regions or genes are known to be selected in domesticated chickens, such as *TSHR* (affecting reproductive behaviors) and the *BCDO2* (for yellow skin) as have been discussed in a number of previous studies [4, 7, 44]. These genes, however, were not picked up in our study as we applied Fst-based approach for detection of selection footprints between meat-type (broiler) and white-egg layer lines. However, both regions were checked in more detail in both lines. In the *TSHR* and *BCDO2* genes, there were 812 and 187 SNPs, respectively and 57 and 23 INDELs, respectively. Most of these variants were fixed (homozygous) in both lines, especially in the *BCDO2* gene, showing that these selection signatures are clearly present in both chicken lines.

### Overlap of selection signatures with known QTLs

In order to gain confidence in detecting selection signatures and linking these regions to potential phenotypes, we compared the identified selection signature regions with known QTL regions according to the Animal QTLdb [21].

Approximately 97% of the selection signature regions overlapped with one or more QTLs for different traits. This provides an independent support for the validity of the selection signature regions detected in this study. These QTLs corresponded to 190 traits related to fat deposition, growth, carcass, egg production and quality, bones, blood parameters, and organs, among others.

In addition, a statistical test was performed to check if overlaps of the selection signature regions with QTLs were significant and not just by chance. This comparison revealed that a higher proportion of the putative signature regions identified in this study were located in QTL regions associated with relevant traits in chicken, and should be further investigated.

The results showed that almost all the selection signature regions overlapped with different QTLs, providing additional confidence of the putative selection regions identified, and also may be an indication of genetic correlation between traits due to pleiotropy.

### SNP and INDEL validation

Availability of variant information from previous NGS studies and high throughput genotype data from 600 K arrays offer an excellent opportunity to carry out cross-study and cross-platform (NGS vs array) comparisons to achieve greater confidence in the variant and genotype calls. We took this opportunity to validate SNPs and INDELs detected in the present study.

SNPs were validated by comparing NGS data against two datasets: 15 M SNPs detected in a previous study by Gheyas et al. [7] and SNP data from 600 K Affymetrix® Axiom® HD genotyping array from two chickens.

We validated more than 78% of our SNPs by comparison with the catalog from Gheyas et al. [7]. This provides a strong validation of the SNPs detected in our study.

In addition, we observed high levels of concordance with 600 K SNP array data. We noticed a slightly higher percentage of genotype concordance for homozygous SNPs than for heterozygous ones, which were also observed previously in a study with bovine variants [61].

The majority of the discordant genotypes checked had poor genotype quality in our NGS data even though their variant qualities were high. A possible explanation of this could be that the sequencing coverage was not sufficient in these cases for accurate prediction of the genotype.

We observed low error rates in our study (considering the non-concordance of genotypes between NGS and array data for SNPs), showing that alignment and SNP calling were efficient.

The 600 K array allowed us to validate a large number of functional variants, intolerant non-synonymous and stopgain/loss SNPs, thereby providing confidence in their detection.

Moreover, INDELs identified in this study were also validated by comparison with two major studies [8, 22], which have generated the largest catalogs of INDELs in chicken. We observed that 77% of the INDELs reported in this study were common with the two previous studies evaluated, and 62% had the same alleles. Similar results were observed before based on the same type of comparison [8]. The lower percentage of concordance, when allele information was considered, is an indication that the INDEL alleles differ among different chicken populations, probably because a large proportion of INDELs actually consists of microsatellite like tandem repeats. This comparison, with the two major INDEL studies in chicken [8, 22], shows that the methodologies used in this study were efficient for detecting and filtering INDELs, and ~ 23% of novel INDELs were detected.

## Conclusions

In this study, we performed a genome-wide identification and characterization of genetic variations, and found putative genomic footprints of selection from two different chicken lines, a white egg layer and a meat-type (broiler) line, which have been under multi-trait selection in Brazilian climatic and nutritional conditions for more than 20 years. Approximately 15 million genetic variants were identified. Of which, over 10 thousand are expected to alter protein functions. Important pathways related to lipid metabolism, growth, and reproductive traits were detected from the analysis of SNPs and INDELs, especially the line-specific variants.

Fst-based analyses were used to identify putative regions of selection showing population differentiation. This approach revealed a number of highly plausible candidate genes and pathways under selection for fat metabolism, growth, reproduction, and cardiac development. Moreover, this is the first study to utilize genome-wide INDEL variants in chicken to identify selection signatures, showing high level of correlation in results between the SNP and INDEL based data.

To minimize the effect of genetic drift, different approaches were applied to identify high confidence regions of putative selection. However, despite the effort to avoid detecting regions caused by random drift, some of the regions detected in this study may represent false positives, especially the ones that did not overlap with genes having known phenotypic association with QTLs. Further analysis in different populations would be required to confirm the accuracy of those regions.

In summary, this study provides novel insights into selection footprints that can help elucidate the functional mechanisms underlying important phenotypic traits relevant to broiler and layer chicken lines. In addition, we have presented a detailed genetic catalog of variants both line-specific and common from two Brazilian broiler and layer chicken lines that can be used for future genomic studies involving association analysis with relevant phenotypes, and consequently, facilitate the identification of causative mutations in chicken, and an important resource for marker-assisted or genomic selection for important traits in chicken.

## Methods

### Genome re-sequencing of experimental chicken lines

Twenty eight chickens from two different experimental lines with pedigree control and multi-trait selection by Embrapa Swine and Poultry National Research Center (Brazil) were sequenced: 14 individuals from a paternal broiler line called TT (7 females and 7 males), and 14 from a layer line called CC (7 females and 7 males). Previously, these two experimental lines were used to generate a reciprocal F_2_ Resource population for QTL mapping studies [13, 14].

The broiler line (TT), originating from the White Plymouth Rock, New Hampshire and White Cornish breeds, has been selected since 1992 for body weight, feed conversion, carcass and parts yields, chick viability, fertility, hatchability of fertile eggs, and reduced fat deposition and metabolic disorders [15]. On the other hand, the white-egg layer line (CC), originating from the White Leghorn, has been selected since 1989 for egg production, egg weight and quality, chick viability, feed conversion, sexual maturity, fertility, hatchability of fertile eggs, and decreased body weight [15]. Chickens were selected based on their performance for the traits mentioned above, and each line was raised under specific feeding regimes [16].

Each chicken was sequenced individually using a paired-end protocol in the HiSeq2500 sequencer (Illumina) with paired read length of 101 bases. Further details on library preparation and sequencing can be obtained from Moreira et al. [23].

### SNP and INDEL identification and filtration

First, the quality of the sequencing reads was checked with FastQC tool [62]. Read quality trimming was performed using SeqyClean tool (v.1.3.12) [63] to select reads with average Phred score quality ≥ 24 and minimum length of 65 bp. The reads were aligned against the Gallus_gallus-4.0 chicken reference genome [64] using Bowtie2 v.2.1.0 [65]. PCR duplicates were removed using Picard [66] (v.1.112). Then, SNP and INDEL identification was performed with SAMtools v.1.2 [67] using the *mpileup* option with mapping and base qualities (Phred score) ≥ 20. All 28 sample BAM files were analyzed together to improve the variant calling in low coverage regions. After the initial calling, different filtration criteria were applied to reduce the number of false-positives and to avoid copy number variation regions. The filtration criteria included (1) Phred-based variant quality score of at least 40; (2) minimum depth of coverage at the variant site of 5; (3) maximum depth of coverage of not more than mean coverage plus 3 standard deviations; (4) variant supported by both forward and reverse strands (at least one read on the forward strand and one on the reverse strand); (5) variant supported by at least 3 reads, and (6) SNP clusters (> 10 SNPs in 50 bp), and INDEL clusters (gap of 1 bp between two INDELs) were removed.

A SNP/INDEL was referred to as homozygous when only a non-reference allele was observed and heterozygous when both the reference and non-reference alleles were observed. The alternate allele frequencies (AAF) of SNPs/INDELs were calculated with VCFtools v. 0.1.12 [68], and AAF was estimated based on the number of times an alternate allele (SNP or INDEL) appears over all individuals at that site, divided by the total number of non-missing alleles at that site.

### Functional annotation and effect prediction

Sets of unique SNPs and INDELs from the 28 chickens were annotated using Variant Effect Predictor tool v.75 [69] against the gene annotation database from Ensembl (release 71) for chicken. For SNP prediction, we also used the SIFT option from VEP tool [18], which predicts functional effects of SNPs and classifies them either as intolerant (affects protein function) or tolerant (functionally neutral) based on amino acid properties and sequence homology (degree of evolutionary conservation).

### Metabolic pathway analyses

QIAGEN’s Ingenuity® Pathway Analysis (IPA®) software [19] was used with default parameters to find metabolic pathways of genes with relevant biological functions from SNPs and INDELs. First, all genes with putative functional coding variants present in both lines were analyzed together to find a global result. Then, variants, exclusively from broilers or layers, were analyzed separately to find metabolic pathways affected in a particular line. Different sets of variants were utilized, viz. genes containing (1) intolerant SNPs (3746 unique genes); (2) intolerant SNPs exclusively from broilers (1825 unique genes) or layers (1057 unique genes); (3) frameshift and non-frameshift INDELs (1462 unique genes), and (4) frameshift and non-frameshift INDELs exclusively from broilers (406 unique genes) or layers (182 unique genes). IPA computes a p-score (network score) derived from *p*-values and calculated using Fisher’s exact test. A network score of > 3 (*p*-value < 0.001) indicates a > 99.9% confidence that a network is not generated by random chance; however, a network score ≥ 30 was considered for determining significant results. Similar cut-off scores were utilized in previous studies to ensure that only highly significant networks were identified [70, 71].

### Genome-wide scan for selection signatures

The Weir and Cockerham pairwise Fst (Fixation index) method [72] was applied to estimate the genome-wide genetic differentiation between broiler and layer lines. Chromosomes W/Z, unplaced, random, and mitochondrial were not considered in this study. This method was performed using VCFtools v. 0.1.12 software [69] with SNP (*n* = 12,806,643) and INDEL (n = 1,273,210) datasets considered separately and using overlapping windows of 20 kb and a step size of 10 kb. Weighted Fst values were calculated for windows with at least 10 SNPs or 5 INDELs. Windows with the top 1% Fst values were considered as candidates of selection signatures, while the extreme top 0.1% windows were considered candidates with strong evidence of selection. The selection signature regions obtained were annotated against the chicken gene database from Ensembl (release 84). All genes from regions under selection (top 1%) were further analyzed to predict the function of these genes with GeneMANIA prediction server [20] considering human database (chicken not available). Gene enrichment analysis was performed considering Q-values calculated from a FDR test, and pathways with Q-values < 0.1 were considered significant.

### QTL overlap analysis

QTL overlap analysis was carried out using the regioneR package [73]. From 190 traits, a subset of 62 representing the most relevant in poultry were selected for this analysis. A permutation test was performed to evaluate the significant associations between the genomic regions (merged signature regions) and the QTL regions by random permutations (*n* = 1000). In every permutation, the overlap with the QTLs was recomputed based on the total genomic size (Mb) that was overlapped. The observed number of overlaps was compared with the empirical distribution to obtain the *p*-values. A p-value < 0.05 was considered to determine significant associations.

### SNP and INDEL validation

The SNPs and INDELs detected in the present study were compared with variants detected from different populations in previous NGS studies as a means of validation. The filtered SNP set was compared with the 15 M SNPs detected in Gheyas et al. [7], which had generated the largest catalog of SNPs in chicken. Similarly, the filtered INDEL set was compared against the combined dataset from two major recent studies: 883,840 INDELs from Boschiero et al. [8] and 1,343,782 INDELs from Yan et al. [22]. Comparisons were made based on both positions and allelic information.

The accuracy of the NGS-based SNP genotype calls was investigated by comparing sequence data from two chickens against the genotype calls from 600 K Affymetrix® Axiom® HD genotyping array from the same chickens, which have been previously genotyped with the array. The chickens chosen for this analysis had intermediate sequencing coverage of ~ 11 X/chicken.

From the NGS dataset, we compared 13,218,246 SNPs with 11.2 X coverage from the layer and 13,229,324 SNPs with 11.5 X average coverage from the broiler chicken. From the chip dataset, the total of 575,599 (layer) and 574,482 (broiler) SNPs were used, excluding the SNPs located on the linkage groups. In both datasets, the chromosomes analyzed were GGA1–28 and sex W/Z. Both heterozygous and homozygous genotypes were considered for comparison of the two datasets. The comparison was performed based on a methodology previously proposed [61] by calculating two types of concordance: (i) POS-CONC - SNPs with same position in both datasets, and (ii) GEN-CONC- only POS-CONC SNPs with same genotypes between the two datasets. The error rate in genotype call from NGS data was estimated as the percentage of POS-CONC SNPs which had non-concordant genotypes between NGS and array data, i.e. error rate % = [100-(number of GEN-CONC SNPs/number of POS-CONC SNPs)*100].

## Additional files

Additional file 1: A figure with the substitution types of SNPs initially identified from the 28 chickens (broiler and layer chickens combined). (PDF 1108 kb)

Additional file 2: A figure with the proportion of heterozygous and homozygous SNPs (A) and INDELs (B) observed in each individual of layer and broiler lines. (PDF 7169 kb)

Additional file 3: An excel file with the top nine networks from frameshift and non-frameshift INDELs from the layer and broiler lines combined. QIAGEN’s Ingenuity® Pathway Analysis (IPA®) software (http://www.ingenuity.com/) with default parameters were used to find metabolic pathways of genes with relevant biological functions. (XLSX 10 kb)

Additional file 4: An excel file with the top six networks from frameshift and non-frameshift INDELs exclusively from the layer or broiler line. QIAGEN’s Ingenuity® Pathway Analysis (IPA®) software (http://www.ingenuity.com/) with default parameters were used to find metabolic pathways of genes with relevant biological functions. (XLSX 10 kb)

Additional file 5: An excel file with the common putative genes under selection from SNP and INDEL based analyses. (XLSX 31 kb)

Additional file 6: An excel file with enrichment analysis results including the enriched GO terms from genes located in top 1% regions under selection (SNP dataset). The enrichment analysis was performed with GeneMANIA prediction server considering human database. (XLSX 9 kb)

Additional file 7: An excel file with enrichment analysis results including the enriched GO terms from genes located in top 1% regions under selection (INDEL dataset). The enrichment analysis was performed with GeneMANIA prediction server considering human database. (XLSX 9 kb)

Additional file 8: An excel file with the intolerant SNPs located within genes under selection (top 1%). (XLSX 11 kb)

Additional file 9: An excel file with the SNP validation comparison results showing the number and percentages of SNPs with same position (POS-CONC) and genotype (GEN-CONC) from broiler and layer chickens of NGS and chip dataset. (XLSX 9 kb)

## Abbreviations

AAF: Alternate Allele Frequency
FLS: Fatty liver hemorrhagic syndrome
GEN-CONC: SNPs with same genotypes
GGA: *Gallus gallus*
GH: Growth hormone
GWAS: Genome-Wide Association Study
INDEL: Insertions and Deletions
IPA: Ingenuity Pathway Analysis
LH: Luteinizing hormone
NGS: Next Generation Sequencing
PCR: Polymerase Chain Reaction
POS-CONC: SNPs with same positions
PRL: Prolactin
QTL: Quantitative Trait Locus
SNP: Single Nucleotide Polymorphism
TS/TV: Transition/Transversion
