# Supplementary figures and images for: Genome-wide characterization of genetic variants and putative regions under selection in meat and egg-type chicken lines
Source: BMC Genomics. 2018 Jan 25;19:83. doi: 10.1186/s12864-018-4444-0 (PMC5785814; doi:10.1186/s12864-018-4444-0)

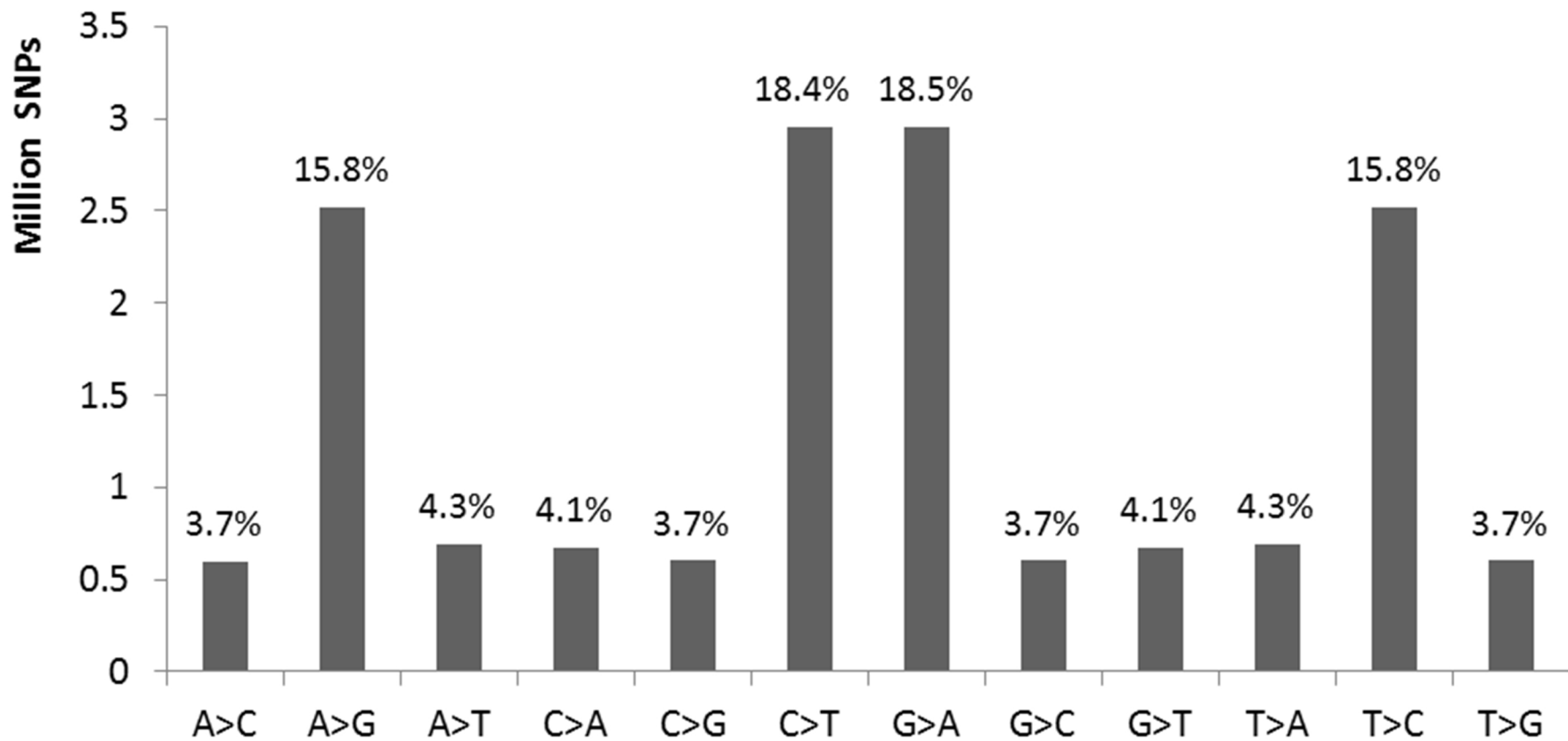

Supplement: Supplementary file 1 — A figure with the substitution types of SNPs initially identified from the 28 chickens (broiler and layer chickens combined). (PDF 1108 kb) [file 12864_2018_4444_MOESM1_ESM.pdf]

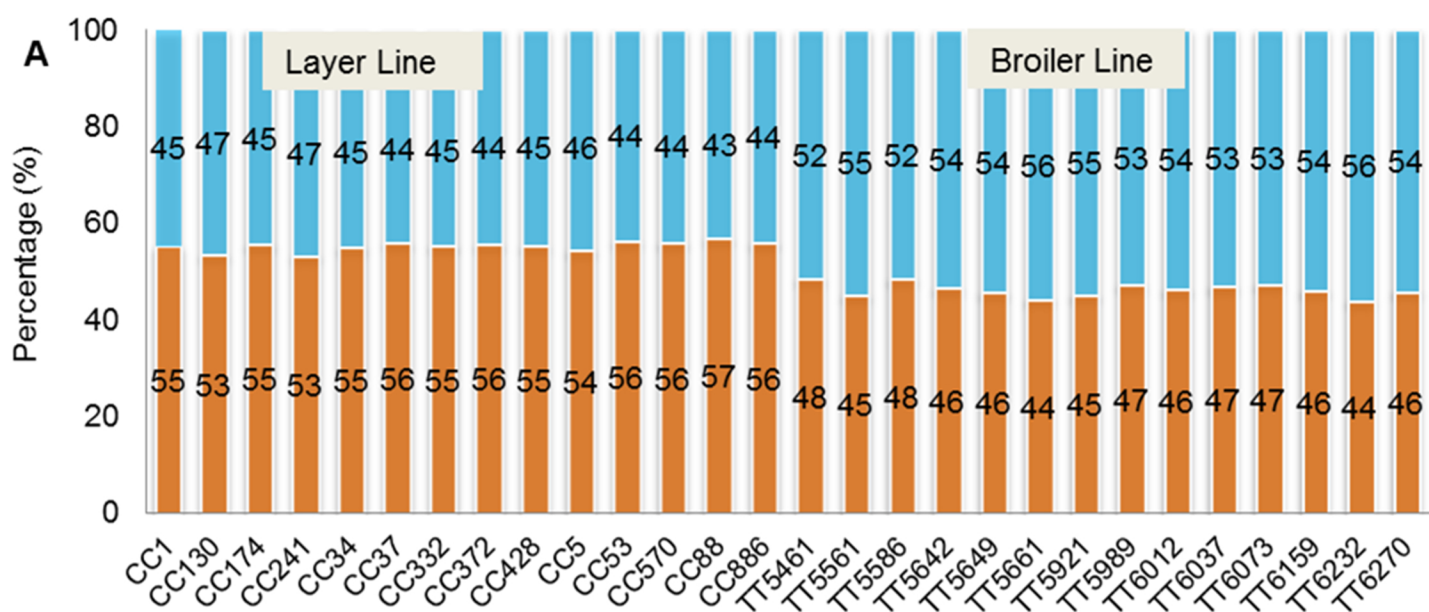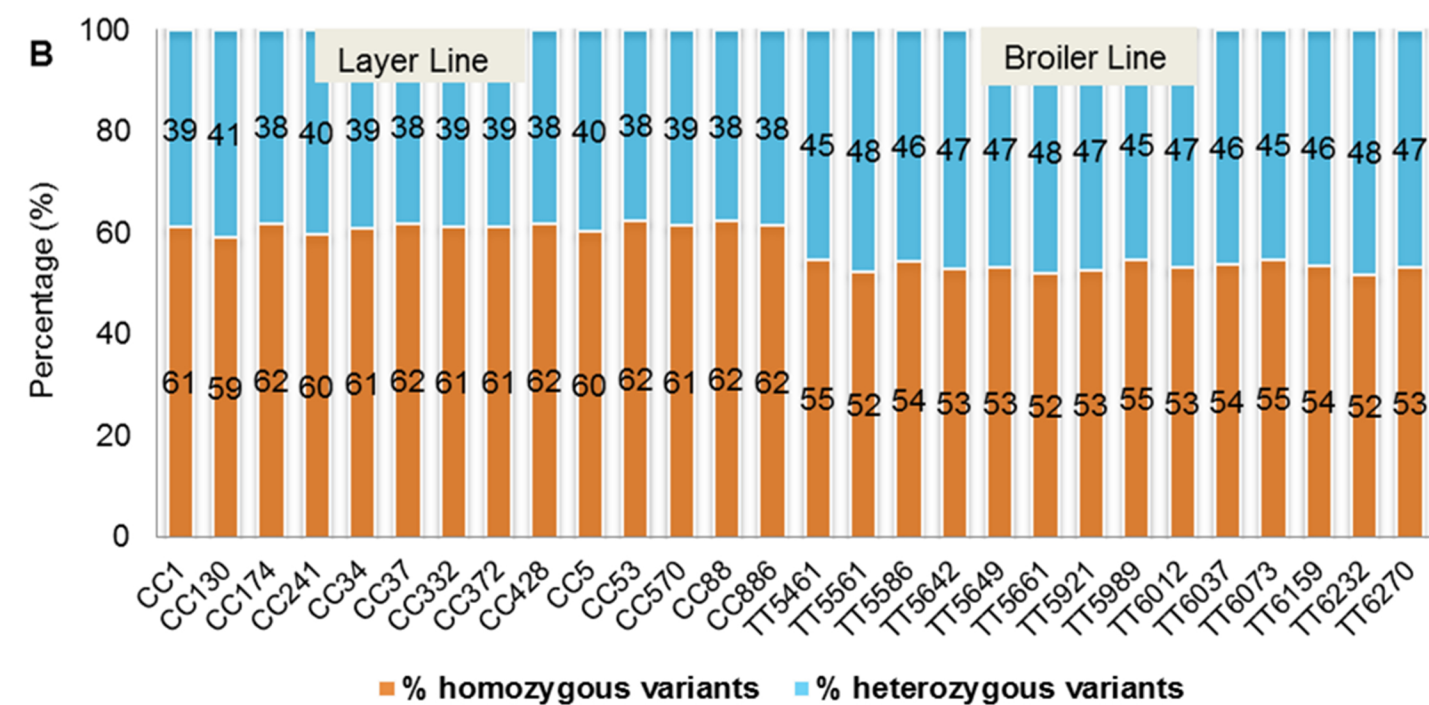

Supplement: Supplementary file 2 — A figure with the proportion of heterozygous and homozygous SNPs (A) and INDELs (B) observed in each individual of layer and broiler lines. (PDF 7169 kb) [file 12864_2018_4444_MOESM2_ESM.pdf]
